# Relative Susceptibility of Brassicas to Cabbage Maggot (Diptera: Anthomyiidae) Infestation

**DOI:** 10.3390/insects14050411

**Published:** 2023-04-26

**Authors:** Shimat V. Joseph

**Affiliations:** Department of Entomology, University of Georgia, 1109 Experiment Street, Griffin, GA 30223, USA; svjoseph@uga.edu

**Keywords:** *Delia radicum*, broccoli, cauliflower, Central Coast of California, Salinas Valley

## Abstract

**Simple Summary:**

Cabbage maggot is a devastating pest of cole crops such as broccoli, cauliflower, Brussels sprout, cabbage, and turnip in the Central Coast of California. Organic and conventional growers in the region have limited access to non-chemical management options. To develop a trap crop, understanding the relative susceptibility of brassicas to cabbage maggot is essential. Thus, brassicas were evaluated as companion plants with broccoli and lettuce after exposure to cabbage maggot flies. In 2013 and 2014, cabbage maggot flies were observed to lay more eggs at the base of turnip plants than broccoli. The larval feeding damage was greater on turnip than on broccoli. Lettuce (a non-brassicaceous crop) was included in the experiment to determine if it could suppress cabbage maggot attacks on broccoli. Lettuce did not reduce cabbage maggot infestation on broccoli after planting side-by-side. Additionally, the number of eggs and larval feeding damage were lower in cauliflower than in broccoli. Cabbage maggot infestation on cabbage was not different from that on broccoli. This study suggests that turnip should be further evaluated by planting on border rows of fields, or as an intercrop, to manage cabbage maggot in broccoli in the Central Coast of California.

**Abstract:**

Cabbage maggot, *Delia radicum* (L.) (Diptera: Anthomyiidae) is a serious pest of *Brassica* such as broccoli (*Brassica oleracea* var. *italica* Plenck) and cauliflower (*B. oleracea* L. var. *botrytis*) in California’s Central Coast. Since there are limited non-chemical options available for growers to manage *D. radicum*, there is an urgent need to develop alternative tactics. The objective of this study was to determine the effects of side-by-side plantings of turnip (*Brassica rapa* var. *rapa* L.), lettuce (*Lactuca sativa* L.), cauliflower, and cabbage (*B. oleracea* L. var. *capitata*) with broccoli on *D. radicum* infestation. In 2013 and 2014, the experiments were conducted in Salinas, California. Significantly greater numbers of eggs and larval feeding damage were found on turnip compared with broccoli. Lettuce (Asteraceae), a non-*Brassica* crop, was compared with broccoli; however, lettuce did not reduce oviposition or larval feeding damage on broccoli. The larval feeding damage on cauliflower was significantly lower than on broccoli when planted side-by-side. The effects on cabbage were not significantly different from broccoli in terms of oviposition and larval feeding damage. This new information generated from the Central Coast of California will be further utilized to develop a trap crop to effectively tackle the *D. radicum* problem in *Brassica* fields.

## 1. Introduction

Cabbage maggot, *Delia radicum* (L.) (Diptera: Anthomyiidae), is a destructive insect pest of brassicaceous crops worldwide [1,2] and in the Central Coast of California [3,4]. In the Central Coast of California (Monterey, Santa Cruz and San Benito counties in California, USA), broccoli (*Brassica oleracea* var. *italica* Plenck) and cauliflower (*B. oleracea* L. var. *botrytis*) are the important *Brassica* crops that are affected by *D. radicum*. In the past few decades, these *Brassica* crops have been consistently ranked among the top ten crops in the Central Coast of California [5,6]. Broccoli and cauliflower crops are valued at >USD 465 million and grown in >23,204 ha in Monterey County [6]. Broccoli is organically produced on ~2942 ha in the region [6]. Other brassicas, such as cabbage (*B. oleracea* L. var. *capitata*), Brussels sprouts (*Brassica oleracea* L. var. *gemmifera*), and turnip (*Brassica rapa* var. *rapa* L.), are also produced in the region. Similarly, lettuce [*Lactuca sativa* L. (Asteraceae)] is a major crop, and was valued at ~ USD 1.2 billion in 2021 [6]. Most vegetable growers rotate lettuce with broccoli or cauliflower during the growing season, although there are many dedicated *Brassica* growers. *Brassica* crops are grown throughout the year, including in the winter. *Delia radicum* poses a persistent and year-long threat to *Brassica* crops in the Central Coast region [4]. Moreover, moderate temperatures during the summer and winter in the region favor *D. radicum* populations [3,7]. *Delia radicum* does not infest lettuce, although other *Delia* spp., such as seedcorn maggot, *Delia platura* (Meigen), occasionally infest lettuce roots [8]. *Delia radicum* infestations can cause widespread crop loss, depending on the infestation timing relative to the crop phenology and densities of *D. radicum* under attack [9,10]. *Delia radicum* larvae feed on roots and girdle the tap root system, causing wilting, yellowing of foliage, and eventual plant mortality [10]. These feeding damages directly affect the yield of broccoli florets (SVJ unpublished data). Growers in the region spend USD 6252 per ha until the broccoli harvest [11]. Severe infestations can cause 100% crop loss ([9] as shown in Figure 3 of Joseph, 2013). It is common to find 20–40% infestation at any time of the year in the Central Coast of California, which can incur crop losses of up to USD 2500 per ha. Pest control advisors spend USD 939 per ha for insect and disease management for broccoli, including the cost of managing cabbage maggots [11]. Broccoli and cauliflower are important rotational crops for lettuce producers, and the market value for brassicas tremendously fluctuates through the year. Any crop loss to *D. radicum* infestation will affect crop yield and increase management costs. Thus, it is critical to manage *D. radicum* as it can cause serious economic losses to growers in the region [4,9].

*Delia radicum* adults invade newly planted *Brassica* plants in the Central Coast of California [4]. The adults make multiple contacts with the surface of the *Brassica* foliage, and non-volatile compounds on the leaves trigger successful oviposition at the base of the stem in the soil [1,12]. The eggs hatch, and the young larvae feed on the root system. As a standard practice in the Central Coast of California, seeds of broccoli are directly planted into soil beds in two seed lines. *Delia radicum* oviposition is observed beginning three weeks after seedling emergence [4]. Cauliflower is a transplanted crop, where *D. radicum* oviposition is observed as early as two weeks after transplanting [13]. *Delia radicum* infestation is also enhanced on broccoli plants when plant residues from the previous crop are inadequately decomposed [14]. The late instar larvae of *D. radicum* extensively feed on the taproots of these plants. *Delia radicum* pupate in the soil, and the emerging adults either re-infest the plants in the same field or disperse to newly planted fields. Since various crops, such as *Brassica*, lettuce, etc., are steadily produced uninterrupted in ~2.8–4.1 ha blocks, and these crops are at various phenological growth stages at any given time in the region, it is easier for *D. radicum* adults to find *Brassica* hosts for infestation and continue their generations. The maturity and growing period of *Brassica* depend on the season and crop species.

The management of *D. radicum* is primarily accomplished using insecticides [10,15,16] sprayed at the correct time for crops planted as seeds [16], seeds pretreated with insecticide (Joseph unpublished data) [15], or by soil drench application for transplanted crops [13]. However, growers seeking *D. radicum* management using organic tools and non-chemical approaches have limited options. In addition, high concentrations of organophosphates [17] and pyrethroids, such as zeta-cypermethrin and lambda-cyhalothrin [18,19], were detected in the surface waters of the Salinas River. Studies showed that these levels of insecticide residues in surface water threaten nontarget organisms and the public through contaminated water; thus, stringent regulations have been implemented [20,21]. These organophosphates are no longer recommended for *D. radicum* management, but pyrethroids are still recommended [10]. Thus, there is a critical need to develop non-chemical options for *D. radicum* management.

**Figure 1:**
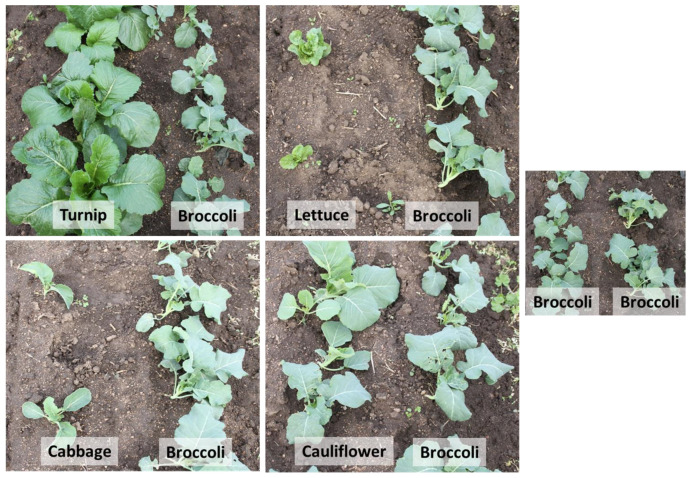
The diagram shows the side-by-side planting of broccoli in one seed line, and an alternate host planted in the other seed line. The seeds were initially planted tight; then, around four weeks later when seedlings emerged, the seedlings were thinned at 7.6, 30.5, 30.5, 30.5, and 15.2 cm spacings for turnip, lettuce, cauliflower, cabbage, and broccoli plants, respectively.

As an alternate approach, trap cropping, has been shown to be an effective approach to intercept invading pests and reduce infestations on the main crop by planting a more preferred trap crop [22,23]. The trap crop restricts or limits the movement of the pests on the main crop and reduces feeding damage. The trap crop technique is effectively utilized by spatially planting along the edges of the field, assuming that the pest population develops on resources that are outside the field [23]. Sometimes, growers take advantage of companion cropping or intercropping for pest management, as the pest is preferentially attracted to one host [24,25]. Trap crops are also temporally planted to destroy pest habitats, such as relay cropping, and have been examined for *D. radicum* management in *Brassica* [26]. However, understanding the relative or no preference or susceptibility of *Brassica* species to *D. radicum* infestation is important to develop a trap crop. Previously, turnip was found to be more attractive to *D. radicum*, as more eggs were laid by the females under turnip than broccoli. [27]. Since many *Brassica* and lettuce crops are grown in the Central Coast of California, a comparative susceptibility study of these major crops to *D. radicum* infestation will be valuable information to develop an effective trap crop. Since broccoli is an important crop by value and hectarage in the Central Coast of California, this study was centered around broccoli as a main crop. Thus, the major objective of this study was to determine the relative susceptibility of various *Brassica* crops, such as cabbage and turnip and lettuce hosts, to *D. radicum* oviposition and severity of damage when direct-seeded and grown side-by-side with broccoli (as shown in Figure 1). The knowledge of the relative susceptibility of various *Brassica* hosts and lettuce to *D. radicum* will help develop an effective trap crop for *Brassica* production in the Central Coast of California.

## 2. Materials and Methods

### 2.1. Study Site, Plants, and Insects

In 2013 and 2014, the experiments were conducted at the United States Department of Agriculture, Agriculture Research Station, Spence Research Farm (36.624302, −121.545992) in Salinas, California. The 8161 m^2^ of land was prepared after tilling and leveling. There were 82 m × 101.6-centimeter (long: wide) beds prepared. One side of the field had a road, and the other faced other fields. The nearby field in 2013 had broccoli planted; in 2014, adjacent fields had no plants planted. In 2013, strawberry (*Fragaria ananassa* Duchesne) was planted 550 m away on the same adjacent ranch. Many weeds, such as shortpod mustard [*Hirschfeldia incana* (L.) Lagr.-Foss.] and perennial wall rocket [*Diplotaxis tenuifolia* (L.) DC] were present along the fences, ditches, and roadsides. These weeds were not managed. 

The seeds of turnip (‘Tokyo’, Sakata Seed America, Inc., Morgan Hill, CA, USA), cauliflower (‘Symphony’, Syngenta/Rogers Vegetable Seeds, Downers Grove, IL, USA), cabbage (‘Gazelle’, Bejo Seeds, Oceano, CA, USA), broccoli (‘Legacy’, Semenis, St. Louis, MO, USA), and lettuce (‘Tehama’, Paragon Seeds, Salinas, CA, USA) were purchased for the experiments. These seeds were widely planted in the Central Coast of California. The seeds were stored at room temperature (22 °C). The *D. radicum* population was naturally present in the area. In 2013 and 2014, the plants were naturally infested with *D. radicum,* as many neighboring *Brassica* fields in the area were infested with *D. radicum* larvae. The experiments were initiated during the mid-to-late summer, as the *D. radicum* population size typically increases by late summer in the Central Coast of California every year.

### 2.2. Experimental Design

The seeds of host plants were planted on to 101.6-centimeter-wide beds with two seed lines, where one seed line was planted with an alternate host, such as turnip, lettuce, cauliflower, cabbage, or broccoli, and the other seed line was planted with broccoli (Figure 1). It was a side-by-side planting of the alternate host along with broccoli. In 2013, the five treatments were as follows: (1) broccoli:turnip, (2) broccoli:lettuce, (3) broccoli:cauliflower, (4) broccoli:cabbage, and (5) broccoli:broccoli. In 2014, cabbage was not included in the experiment based on the analysis of 2013 data; thus, the four treatments were (1) broccoli:turnip, (2) broccoli:lettuce, (3) broccoli:cauliflower, and (4) broccoli:broccoli. The treatments were replicated five times in a randomized complete block design. Since soil moisture, fertility, or *D. radicum* infestation, etc., could vary from various sides of the field, the treatments were blocked from one side of the field to the other. The plot size was ten 101.6 cm × 9.1 m (wide: long) beds. The seeds of the hosts were planted at high density using a tractor-mounted seeder. A month after planting seeds, when all the seeds germinated, the seedlings were hand-thinned a week after seedling emergence, in order to maintain the commercially acceptable spacing between seedlings for the respective hosts. The spacings for turnip, lettuce, cauliflower, cabbage, and broccoli plants were 7.6, 30.5, 30.5, 30.5, and 15.2 cm, respectively. In 2013, host seeds were planted on 14 September, whereas in 2014, seeds were planted on 18 July. The plants were irrigated using overhead sprinklers that were installed after planting the seeds. The beds were irrigated at 3 d per week for 4 h per d until the seedlings were established. The plants were grown following commercial practices for *Brassica* and lettuce production in the region; however, no insecticides or fungicides were applied. A pre-emergence herbicide, dimethyl tetrachloroterephthalate (Dacthal^®^ flowable [54.9%], AMVAC, Los Angeles, CA, USA) at 4.5 L per 0.41 ha, and Bensulide [S-(O,O-diisopropyl phosphorodithioate) ester of *N*-(2-mercaptoethyl) benzenesulfonamide] (Prefar^®^ 4-E [46%], Gowan Company, LLC, Yuma, AZ, USA) at 3.8 L per 0.41 ha, were tank-mixed and applied to all of the beds, except where lettuce seeds were planted. These pre-emergent herbicides were compatible with *Brassica* crops, but not compatible with lettuce. The weeds on broccoli:lettuce plots were hand-picked every week. To ensure uniform germination, phosphoric acid was applied on the beds at 132.5 L per 0.41 ha as an anti-crusting agent before planting the seeds. Fertilizer (28-0-0- 5; N-P-K- S) was side-dressed at 280.6 L per ha a month after planting the seeds. Ammonium sulfate was side-dressed two weeks later at 336.3 kg per ha.

### 2.3. Sampling and Evaluation

*Delia radicum* eggs were oviposited on or in the soil surface within a ~10 cm diameter of the plant base [1]. The soil was sampled from a 10 cm diameter, 2.5 cm deep, using a hand trowel from ten plant bases per plot (replication). Eggs were extracted from the soil using a floatation method. The soil samples from ten plants were combined, the combined sample per plot (replication) was added to a plastic pail, and 500 mL of tap water was added to the soil. The soil was agitated for 1 min so that the *D. radicum* eggs, if present, could float to the surface of the water. The water was decanted through a 65% black cotton fabric (Creative Cuts Palencia, Pensacola, FL, USA). All of the soil samples were subjected to the floatation method two times, in order to ensure that most of the eggs were recovered from the soil samples. To determine *D. radicum* root damage, ten plant roots from broccoli and alternate hosts were randomly sampled from each plot (replication) every week, starting a week after plant emergence. The sampling involved carefully uprooting the plants with minimal root damage. Most of the *D. radicum* feed injury on roots was observed on the tap root or swollen stem of the turnip under the soil. The plant root samples were evaluated for the severity of *D. radicum* feeding damage on roots. The scale system used in the current study was described in Joseph (2016). The root or turnip stem was scored as follows: 0, not infested; 1, infested or >90% root hairs present; 2, 80–90% root hairs present; 3, 70–79% root hairs present; 4, 60–69% root hairs present; 5, 50–59% root hairs present or <25% root destroyed; 6, 40–49% root hairs present or 25–49% root destroyed; 7, 30–39% root hairs present or 50–74% root destroyed; 8, 20–29% root hairs present or >75–89% root destroyed; or 9, no root hairs present or >90% root destroyed. The scale scores from 10 plant roots were averaged for each replication. Foliage samples were collected beginning the second week after seedling emergence in 2013 for 27 September; 4, 11, 17, 24, and 31 October; 7 and 15 November; and in 2014 for 30 July; 6, 11, 20, and 27 August; and 2, 8, and 16 September. In addition, 10 *Delia* spp. larvae were extracted from the roots of 10 plants and were identified as *D. radicum* [28].

To monitor the natural populations of adult *Delia* spp. during the experiment, four 18 × 14 cm yellow sticky cards (Alphascent Inc., West Linn, OR, USA) with sticky surfaces on both sides were placed at four corners of the experimental site. The yellow sticky card traps were serviced twice a week to reduce the overcrowding of flies, but flight activity was reported at weekly intervals. The yellow sticky cards were exposed to *Delia* spp. for 3 and 4 d intervals during the deployment week, and were serviced in 2013 on 26 September; 1, 10, 17, 24, and 31 October; 7, 14, 21, and 28 November; and 5 December; and in 2014 on 31 July; 7, 14, 21, and 28 August; and 4, 11, and 18 September.

### 2.4. Statistical Analyses

All of the data analyses were performed in SAS [29]. Since the objective of the study was to determine how the alternate hosts directly compared with broccoli, the numbers of *D. radicum* eggs captured from alternate hosts were subtracted from captures from broccoli for each sample date. For analysis purposes, the difference data were optimized by adding the lowest negative data point to all the data points, in order to generate a positive data set. Thus, 314 and 6 were added to each data point for the 2013 and 2014 datasets, respectively. This process converted the entire dataset to positive territory. The optimized 2013 *D. radicum* egg data were subjected to analysis of variance (ANOVA) using the PROC GLMMIIX procedure in SAS, with log-link and negative binomial distribution. The treatment and block were the fixed and random effects, respectively. The means were separated using the Tukey–Kramer method (*α* = 0.05). The 2014 *D. radicum* egg data did not converge after using the PROC GLMMIIX procedure in SAS. Thus, the data were subjected to ANOVA using the PROC GLM procedure in SAS after log transformation (ln[x + 2]). The normality of the residuals was checked using the PROC UNIVARIATE procedure in SAS. The treatment and block were the fixed and random effects, respectively. The means were separated using Tukey’s HSD method (*α* = 0.05). Since the goal was to determine the effects of the alternate hosts on broccoli in a direct comparison and *D. radicum* egg oviposition varied by sampling week, the ANOVA was performed at the sampling week. To compare the specific alternate host with broccoli, paired *D. radicum* egg data were analyzed using the PROC TTEST procedure in SAS when eggs were abundantly collected. Furthermore, *t* tests were conducted between total numbers of eggs collected from broccoli and broccoli plus alternate host. The *t*-test for the pooled method was used to determine the levels of significant differences.

The severity of *D. radicum* larval feeding damage for the 2013 and 2014 data was optimized with the same procedure as that used for the egg data. The severity of damage data of broccoli was subtracted from the severity of damage data of alternate hosts for each sample date. The difference data were optimized by adding the lowest negative data point to all of the data points to generate a positive data set. Thus, the score values of 4.4 and 1.5 were added to each data point for the 2013 and 2014 datasets, respectively. The optimized 2013 and 2014 severity of damage data were subjected to ANOVA using the PROC GLM procedure in SAS after log transformation (ln[x + 2]). To compare the specific alternate host with broccoli, the paired severity of damage data was analyzed using the PROC TTEST procedure in SAS. The *t*-test for the pooled method was used to determine the levels of significant differences. Means and standard errors for the nontransformed *D. radicum* eggs and the severity of larval feeding data were calculated using the PROC MEANS procedure in SAS.

## 3. Results

### 3.1. Delia spp. Adults

**Figure 2:**
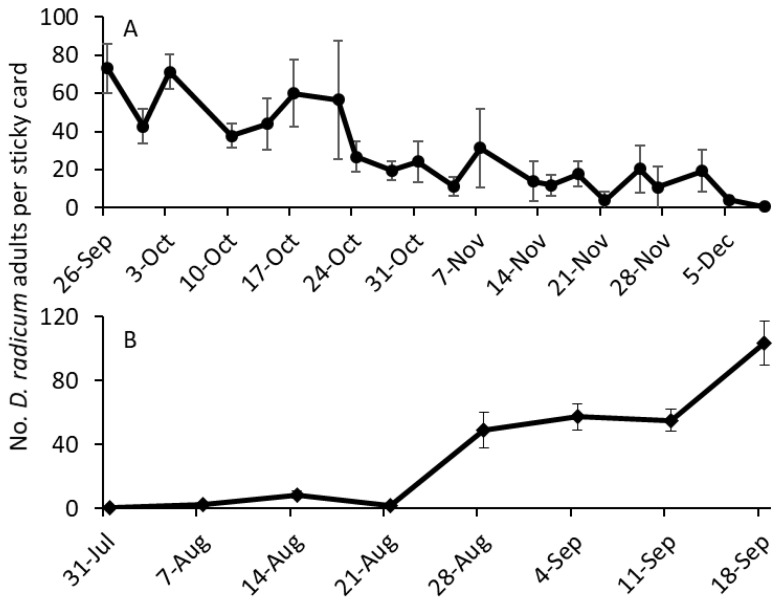
Mean (±SE) captures of adult *Delia* spp. on yellow sticky cards placed at the four corners of the experimental field in (**A**) 2013 and (**B**) 2014.

In 2013 and 2014, *Delia* spp. adults were collected in the experimental plots for the entire study duration (Figure 2). The data showed that adults of *Delia* spp. were present when experiments were conducted. These experiments were conducted from September to December in 2013, and from July to September in 2014, as growers planted *Brassica* crops during this period in the Central Coast of California.

### 3.2. D. radicum Eggs

**Figure 3:**
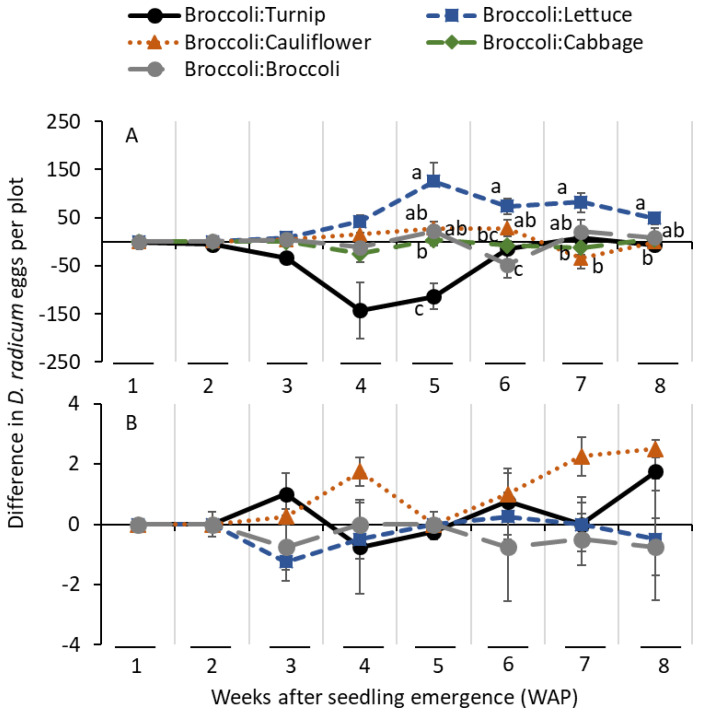
Mean (±SE) differences in *D. radicum* eggs captured on alternate hosts deducted from the captures of *D. radicum* eggs from broccoli (number of eggs from broccoli minus number of eggs from the alternate host) when planted side-by-side in the field conditions in (**A**) 2013 and (**B**) 2014. The same letters within a sampling date were not significantly different among differences in *D. radicum* eggs among alternate hosts (Tukey–Kramer test for 2013 and Tukey’s HSD test for 2014, α = 0.05).

In 2013, *D. radicum* eggs were observed in the soil starting 3 weeks after planting (WAP) (Figure 3A). The differences in the numbers of eggs were not significantly different among alternate hosts at 3 and 4 WAP (Table 1; Figure 3A). At 5 WAP, a significantly lower difference in densities of eggs was collected for the broccoli:turnip treatment than for the broccoli:cabbage treatment, followed by the broccoli:lettuce treatment (Table 1; Figure 3A). At 6 WAP, the difference in eggs was significantly lower for the broccoli:broccoli treatment than for the broccoli:turnip treatment, followed by the broccoli:lettuce treatment. At 7 and 8 WAP, the difference in the number of eggs was greater for the broccoli:lettuce treatment than for the broccoli:cauliflower or broccoli:turnip treatments (Table 1; Figure 3A). In 2014, *D. radicum* eggs were observed in the soil starting 3 WAP (Figure 3B). The differences in eggs among treatments were not significantly different at any of the observation intervals, as low densities of eggs were collected.

**Figure 4:**
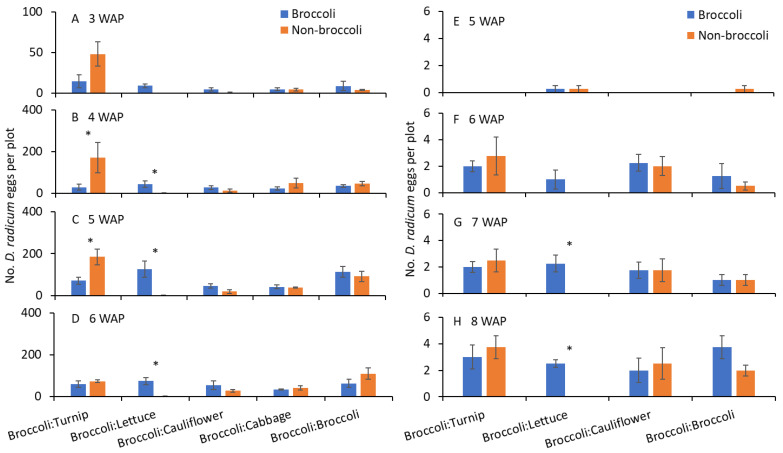
Mean (±SE) number of *D. radicum* eggs collected from broccoli and alternate hosts at (**A**) 3, (**B**) 4, (**C**) 5, and (**D**) 6 weeks after plant emergence in 2013, and (**E**) 5, (**F**) 6, (**G**) 7, and (**H**) 8 weeks after plant emergence in 2014. Asterisks above broccoli and alternate hosts indicate significant differences (*t*-test, α = 0.05).

In 2013, significantly greater numbers of eggs were collected on turnip when compared with broccoli at 4 and 5 WAP (Table 2; Figure 4B,C), although there were no significant differences in the numbers of eggs between broccoli and turnip at 3 and 6 WAP (Table 2; Figure 4A,D). The counts of eggs were significantly lower in lettuce than in broccoli at 4–6 WAP (Table 2; Figure 4B–D); however, when the numbers of eggs collected in broccoli were compared with cauliflower, cabbage, and broccoli, none of the pairs significantly differed for any WAP observations (Table 2; Figure 4A–D). In 2014, the numbers of eggs were significantly lower in lettuce than in broccoli at 7 and 8 WAP, although there were no significant differences in the numbers of eggs collected at 5 and 6 WAP (Table 2; Figure 4G,H). However, when the numbers of eggs collected in broccoli were compared with turnip, cauliflower, cabbage, and broccoli, none of the pairs significantly differed for any WAP observations (Table 2; Figure 4E–H).

In 2013 and 2014, the total numbers of *D. radicum* eggs collected through the experimental period were significantly greater in the broccoli plus alternate hosts (or broccoli) treatments than in the broccoli treatment for all of the pairings except for the broccoli plus lettuce and broccoli pairings (Table 3).

### 3.3. Severity of D. radicum Damage

**Figure 5:**
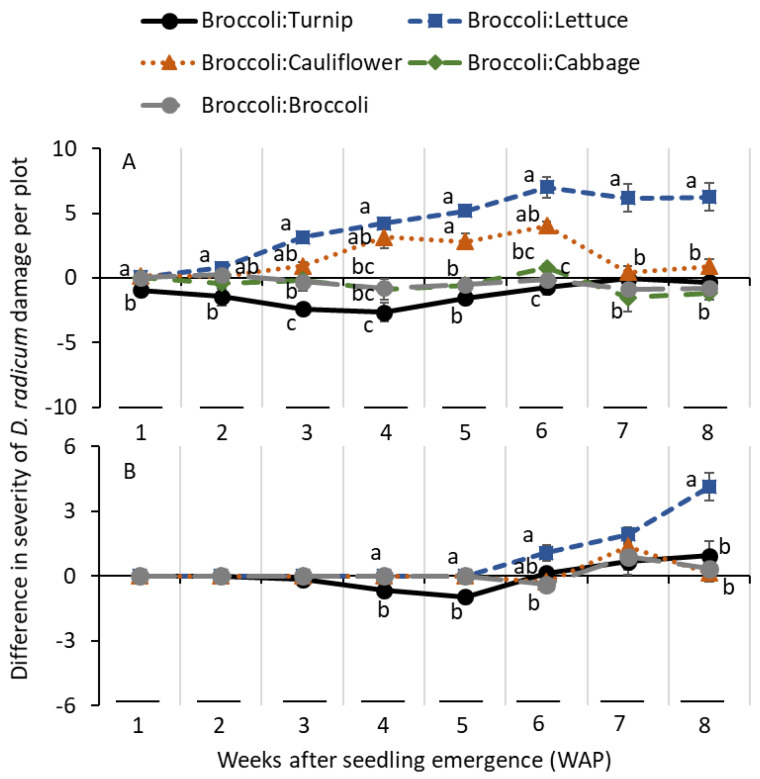
Mean (±SE) differences in severity of *D. radicum* larval feeding damage scores on alternate hosts deducted from the captures of severity of feeding damage from broccoli (severity score from broccoli minus severity score from alternate host) when planted side-by-side in the field conditions in (**A**) 2013 and (**B**) 2014. Same letters within a sampling date were not significantly different among differences in severity of damage scores among alternate hosts (Tukey’s HSD test, α = 0.05).

In 2013, the differences in the severity of damage scores on roots were significantly lower for the turnip treatment than for the lettuce treatment at 1 and 2 WAP (Table 1; Figure 5A). At 3 WAP, the differences in the severity of damage scores were significantly lower for the turnip treatment than for the broccoli treatment, followed by the lettuce treatment. At 4 WAP, significantly lower differences in scores of damage severity were observed for the turnip treatment than for the cauliflower and lettuce treatments (Table 1; Figure 5A). At 5 WAP, the differences in scores of damage severity were significantly lower for the turnip, cabbage, and broccoli treatments than for the cauliflower and lettuce treatments. At 6 WAP, significantly lower differences in severity of damage scores were observed for the turnip, broccoli, and cabbage treatments than for the lettuce treatment. At 7 and 8 WAP, the differences in damage severity scores were significantly greater for the lettuce treatment than for the remaining treatments (Table 1; Figure 5A). In 2014, the differences in damage severity scores were significantly greater for the lettuce treatment than for the remaining treatments at 4, 5, and 8 WAP (Table 1; Figure 5B). There was no significant difference on the differences in severity of damage at 7 WAP (Table 1; Figure 5B).

**Figure 6:**
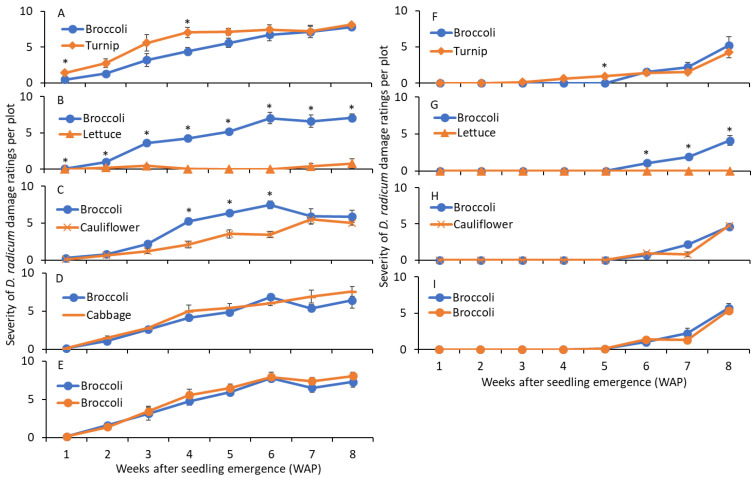
Mean (±SE) severity of *D. radicum* larval feeding damage scores recorded from broccoli and alternate hosts (**A**) turnip, (**B**) lettuce, (**C**) cauliflower, (**D**) cabbage, and (**E**) broccoli up to 8 weeks after plant emergence in 2013, and (**F**) turnip, (**G**) lettuce, (**H**) cauliflower, and (**I**) broccoli up to 8 weeks after plant emergence in 2014. The presence of asterisks above broccoli and alternate host for each sampling date indicate significant difference (*t*-test, α = 0.05).

In 2013, the severity of damage scores was significantly greater on turnip than on broccoli at 1 and 4 WAP, although there were no significant differences in scores between broccoli and turnip at 2, 3, 5, 6, 7, and 8 WAP (Table 4; Figure 6A). For lettuce, the severity of damage scores was significantly lower on lettuce than on broccoli for all WAP observations (Table 4; Figure 6B). The severity of damage scores was significantly lower on cauliflower than on broccoli at 4–6 WAP (Table 4; Figure 6C) and there was no significant difference on the remaining WAP. The severity of damage scores were not significantly different for cabbage and broccoli for any WAP (Table 4; Figure 6D,E). In 2014, the severity of damage scores was significantly greater on turnip than on broccoli at 5 WAP, although there were no significant differences in scores between broccoli and turnip for the remaining WAP (Table 4; Figure 6A). For lettuce, the severity of damage scores was significantly lower on lettuce than on broccoli at 6–8 WAP (Table 4; Figure 6G). The severity of damage scores were not significantly different for cauliflower and broccoli for any WAP (Table 4; Figure 6H,I).

## 4. Discussion

During the early stages of plant development, the turnip was more susceptible to *D. radicum* attack than broccoli. This was evident as more eggs and root damage were observed on the turnip when planted side-by-side with the broccoli. Although a previous study showed that turnip was highly susceptible to *D. radicum* infestations in the Central Coast of California [16], it did not compare turnip and broccoli in a side-by-side setting. *Delia radicum* preferred turnip over broccoli in oviposition [27,30,31], which is consistent with the results in the current study. *Delia radicum* infestation tended to be greater on the edges of the broccoli field than on the interior of the field [4]. Since side-by-side planting of broccoli and turnip showed early *D. radicum* infestation on turnip, this suggests that planting turnip early along the edges of the broccoli field may trap and retain the invading *D. radicum* adults, limit infestations on young broccoli plants, and provide them a better chance to establish. An incidence of *D. radicum* was observed from two to three weeks after emergence of turnip and broccoli seedlings [4,16]. The data show that turnip is more attractive for oviposition than broccoli, which could be utilized to develop turnip as a trap crop for broccoli main crops. Turnip and broccoli are direct-seeded crops in the Central Coast of California; thus, once developed, both conventional and organic growers can utilize a trap crop strategy. An early infestation of *D. radicum* in particular can cause serious damage and effects to broccoli floret yields (SVJ unpublished data). The current study did not evaluate the sizes of turnip and broccoli plants. More research is warranted to determine the effects of planting a few beds of turnip plants along the border of broccoli fields to restrict the advancement of *D. radicum* adults and damage to broccoli. Previously, when a turnip trap crop was evaluated with cauliflower on flea beetle, *Phyllotreta* spp. (Coleoptera: Chrysomelidae), the potential benefits of a turnip trap crop were observed with cauliflower main crops [32], but not with white cabbage [(*Brassica oleracea* L. convar. *capitata* [L.] Alef. var. *alba* DC.)] [33]. Thus, turnip plants should be further investigated as a potential trap crop in broccoli fields on *D. radicum* in the Central Coast of California.

In the current study, *Brassica* hosts were planted using a commercially acceptable plant-to-plant spacing, which varied tremendously among *Brassica* crops and lettuce. The architecture of host seedlings at the early growth stages varied, as turnip plants had wider and longer foliage, followed by broccoli and cauliflower, than lettuce and cabbage (Figure 1). Finch and Collier (2000) suggested an appropriate/inappropriate landings theory, where incoming *D. radicum* were likely to land on plants, depending on the proportional plant and non-plant areas. If the area occupied within plants was mostly composed of non-*Brassica* plants, they are likely to land on non-*Brassica* plants. The trigger for *D. radicum* oviposition is proportional to multiple contacts on nonvolatile compounds on *Brassica* leaves [12]. Once a sufficient dosage via multiple contacts is accumulated during the spiral flight around the plant after landing [34], oviposition is stimulated. The results showed that the densities of *D. radicum* eggs and feeding damages were not lower on broccoli when lettuce was planted side-by-side. Perhaps this result is related to *D. radicum* flies contacting broccoli leaves more than lettuce. Lettuce crops at early development stages had narrower and shorter leaves than broccoli (Figure 1), which decreased the chances of more contact with lettuce than with broccoli. Any contact with non-*Brassica* hosts during the spiral flights may result in incomplete oviposition bouts [26,34,35,36,37,38,39]. The data show that the oviposition of *D. radicum* adults was reduced when lettuce was planted in a relay cropping setting [26]. It is possible that *D. radicum* adults did not make sufficient contact with lettuce during their spiral descent around the plants. It appears that the size of the plants is a critical factor in eliciting a response [40,41]. Moreover, the bare ground space between the beds and plants between the seed lines could have affected *D. radicum* adults’ ability to locate hosts [42]. In commercial settings, planting lettuce seeds earlier than broccoli seeds may be challenging for growers, as they have to coordinate the irrigation timing and operation of farm machinery in wet fields. Transplanting lettuce crop is not a popular practice in the Central Coast of California; however, transplanting larger seedlings could tip the balance, as non-*Brassica* plants occupy most of the field’s green area, reducing *D. radicum* adult contact with *Brassica* hosts and causing poor oviposition.

In contrast, although broccoli and turnip crops were of the same age, they grew at different rates. The leaves of turnip plants were longer and wider than those of broccoli leaves at the early stages of development. Thus, arriving *D. radicum* may have contacted more turnip than broccoli plants, and accumulated the required threshold for oviposition more quickly than on broccoli plants.

The data show that the severity of *D. radicum* feeding damage was lower on cauliflower than on broccoli, although there was no difference in the oviposition. This suggests that the *D. radicum* feeding damage developed more slowly on cauliflower than on broccoli roots. This does not support using cauliflower as a trap crop for broccoli main crops, but rather suggests that broccoli could be developed as a trap crop for cauliflower main crops. Although cauliflower was not directly compared with turnip in the current study, a turnip trap crop has greater potential, and can successfully reduce cauliflower damage. A previous study showed that the flea beetle adults of Phyllotreta spp. were effectively retained on the turnip trap crop from the cauliflower main crop [32]. Similarly, an oilseed rape (*Brassica napus* subsp. *napus*) trap crop was effective in reducing rape blossom beetle, *Meligethes aeneus* (Fabricius) (Coleoptera: Nitidulidae), in the management for a cauliflower trap crop [43]. These studies suggest that having a less attractive host to *D. radicum* infestation as a main crop provides an added value to developing an effective trap crop. In addition, results indicated that no differences in oviposition and root damage between broccoli and cabbage were found, suggesting that cabbage has a limited scope for trap crop development.

## 5. Conclusions

The side-by-side planting of broccoli with turnip showed that oviposition and *D. radicum* larval feeding damage appeared early and significantly more on turnip than on broccoli. This suggests that a turnip trap crop is a promising option, and should be further evaluated in California’s Central Coast production system. The results also showed that companion planting of broccoli with lettuce did not reduce oviposition and feeding damage on broccoli. Perhaps the effects of early versus delayed planting of lettuce should be further evaluated on broccoli, as the current study only evaluated concurrent planting of lettuce and broccoli on *D. radicum* oviposition and larval feeding damage. The data showed that *D. radicum* larval feeding was relatively lower on cauliflower than on broccoli when planted side-by-side.

## Figures and Tables

**Table 1:** Analysis of variance on the difference in the number of *Delia radicum* eggs collected from plant base, and severity of larval feeding damage from broccoli and alternate hosts (such as turnip, lettuce, cauliflower, cabbage, and broccoli) planted side-by-side in 2013 and 2014 experiments.

Week ^a^ after Seedling Emergence	2013						2014					
	Eggs			Severity of Injury			Eggs			Severity of Injury		
	*F*	df	*P*	*F*	df	*P*	*F*	df	*P*	*F*	df	*P*
1 ^b^	1.0	4,12	1.000	7.6	4,12	0.002	- ^d^	-	-	- ^d^	-	-
2	0.1	4,12	0.972	3.1	4,12	0.047	0.0	3,12	0.999	-	-	-
3	- ^c^	-	-	19.5	4,12	<0.001	2.6	3,12	0.099	1.0	3,12	0.426
4	0.9	4,12	0.451	11.9	4,12	<0.001	1.1	3,12	0.377	26.5	3,12	<0.001
5	19.0	4,12	<0.001	28.5	4,12	<0.001	0.3	3,12	0.840	12.3	3,12	<0.001
6	10.5	4,12	<0.001	17.7	4,12	<0.001	0.7	3,12	0.593	5.2	3,12	0.015
7	8.9	4,12	0.001	8.9	4,12	<0.001	2.3	3,12	0.129	0.9	3,12	0.473
8	3.5	4,12	0.042	20.5	4,12	<0.001	1.4	3,12	0.292	8.5	3,12	0.003

^a^ Host seeds were planted on 14 September 2013 and 18 July 2014. ^b^ Samples were collected beginning the second week of seedling emergence in 2013 on 27 September; 4, 11, 17, 24, and 31 October; 7 and 15 November; and in 2014 on 30 July; 6, 11, 20, and 27 August; 2, 8, and 16 September. ^c^ Statistics were not provided because the data did not converge when the ANOVA was performed using the PROC GLIMMIX procedure using SAS. ^d^ ANOVA was not performed because low densities of *D. radicum* were captured in 2014.

**Table 2:** *t*-tests for paired comparisons of the numbers of *Delia radicum* eggs collected from the plant base of alternate hosts (such as turnip, lettuce, cauliflower, cabbage, and broccoli) and broccoli planted side-by-side in 2013 and 2014 experiments.

Week ^a^ after Seedling Emergence	Broccoli:Turnip			Broccoli:Lettuce			Broccoli:Cauliflower			Broccoli:Cabbage			Broccoli:Broccoli		
	*t*	df	*P*	*t*	df	*P*	*t*	df	*P*	*t*	df	*P*	*t*	df	*P*
2013															
3 ^b^	−1.9	6	0.097	- ^c^	-	-	2.1	6	0.085	0.2	6	0.842	0.9	6	0.412
4	−1.9	6	0.103	2.7	6	0.035	1.3	6	0.235	−0.9	6	0.371	−0.9	6	0.395
5	−2.8	6	0.033	3.3	6	0.017	2.1	6	0.086	0.4	6	0.675	0.6	6	0.571
6	−0.9	6	0.422	4.5	6	0.004	1.3	6	0.241	−0.7	6	0.509	−1.4	6	0.222
2014															
5 ^b^	- ^d^	-	-	0	6	1.000	- ^d^	-	-	- ^e^	-	-	−1	6	0.356
6	−0.5	6	0.633	1.4	6	0.207	0.3	6	0.801	-	-	-	0.8	6	0.477
7	−0.5	6	0.620	3.6	6	0.012	0.0	6	1.000	-	-	-	0.0	6	1.000
8	−0.6	6	0.571	8.7	6	<0.001	−0.3	6	0.750	-	-	-	1.9	6	0.114

^a^ Host seeds were planted on 14 September 2013 and 18 July 2014. ^b^ Soil samples were collected beginning the second week after seedling emergence in 2013 on 27 September; 4, 11, 17, 24, and 31 October; 7 and 15 November; in 2014 on 30 July; 6, 11, 20, and 27 August; 2, 8, and 16 September. ^c^ Since the *D. radicum* eggs sampled in 2013 were of low densities from 1 and 2 weeks after seedling emergence and there were no statistical differences beyond 6 weeks after seedling emergence, the statistical analyses are not presented in the table. ^d^ Similarly, in 2014, the densities of eggs collected were of low densities until 4 weeks after seedling emergence. Thus, they are not presented in the table. ^e^ Cabbage treatment was not included in the 2014 experiment.

**Table 3:** *t*-tests for paired comparisons of the total numbers of *Delia radicum* eggs (mean±SE) collected from the bases of broccoli and broccoli + alternate hosts (such as turnip, lettuce, cauliflower, cabbage, and broccoli) when planted side-by-side in 2013 and 2014 experiments.

Treatment Pairs	Total *D. radicum* Eggs ^a^	
	2013	2014
Broccoli	330.3 ± 69.9b	22.5 ± 1.0b
Broccoli + Turnip	965.3 ± 235.9a	53.3 ± 2.6a
*t*	−2.9	−11.1
*P*	0.042	<0.001
Broccoli	438.0 ± 54.1a	20.3 ± 1.9a
Broccoli + Lettuce	443.8 ± 53.7a	20.3 ± 1.9a
*t*	−0.10	1.00
*P*	0.943	0.000
Broccoli	346.3 ± 43.4b	18.0 ± 1.0b
Broccoli + Cauliflower	658.0 ± 82.8a	35.0 ± 2.0a
*t*	−3.33	−7.5
*P*	0.016	<0.001
Broccoli	255.3 ± 45.4b	-
Broccoli + Cabbage ^b^	546.0 ± 94.2a	-
*t*	−2.8	-
*P*	0.032	-
Broccoli	464.8 ± 68.4b	19.5 ± 1.8b
Broccoli + Broccoli	927.8 ± 111.1a	40.8 ± 2.1a
*t*	−3.6	−7.7
*P*	0.012	<0.001

^a^ *D. radicum* eggs collected in 2013 and 2014 experiments were combined. ^b^ Cabbage treatment was not included in 2014 experiment. Same letters between broccoli and broccoli + alternate hosts (or broccoli) indicate not significantly different (*t*-test, α = 0.05).

**Table 4:** *t*-tests for paired comparisons of the severity of *Delia radicum* larval feeding from the foliage of alternate hosts (such as turnip, lettuce, cauliflower, cabbage, and broccoli) and broccoli planted side-by-side in 2013 and 2014 experiments.

Week ^a^ after Seedling Emergence	Broccoli:Turnip			Broccoli:Lettuce			Broccoli:Cauliflower			Broccoli:Cabbage			Broccoli:Broccoli		
	*F*	df	*P*	*F*	df	*P*	*F*	df	*P*	*F*	df	*P*	*F*	df	*P*
2013															
1 ^b^	−3.0	6	0.023	-	-	-	1.5	6	0.178	0.0	6	1.000	0.3	6	0.766
2	−2.4	6	0.055	3.0	6	0.023	0.4	6	0.736	−1.2	6	0.295	−0.8	6	0.482
3	−1.7	6	0.148	8.6	6	<0.001	2.2	6	0.069	−0.7	6	0.520	−0.3	6	0.786
4	−3.0	6	0.023	20.5	6	<0.001	4.9	6	0.003	−0.9	6	0.364	−0.8	6	0.435
5	−1.9	6	0.104	20.7	6	<0.001	4.6	6	0.004	−0.9	6	0.385	−0.8	6	0.412
6	−0.7	6	0.512	8.9	6	<0.001	6.1	6	0.001	−2.2	6	0.071	−0.2	6	0.835
7	−0.1	6	0.951	6.6	6	0.001	0.4	6	0.735	−1.6	6	0.168	−1.1	6	0.302
8	−0.9	6	0.378	7.9	6	<0.001	1.0	6	0.344	−0.9	6	0.390	−0.9	6	0.387
2014															
5	5.0	6	0.002	- ^c^	-	-	-	-		- ^d^	-	-	0.0	6	1.000
6	−0.8	6	0.477	−2.8	6	0.031	1.1	6	0.327	-	-	-	1.6	6	0.152
7	−1.1	6	0.328	−5.8	6	0.001	−2.4	6	0.055	-	-	-	−1.3	6	0.252
8	−0.7	6	0.534	−6.4	6	<0.001	−0.5	6	0.640	-	-	-	−0.6	6	0.587

^a^ Host seeds were planted on 14 September 2013 and 18 July 2014. ^b^ Foliage samples were collected beginning the second week after seedling emergence in 2013 on 27 September; 4, 11, 17, 24, and 31 October; 7 and 15 November; and in 2014 on 30 July; 6, 11, 20, and 27 August; 2, 8, and 16 September. ^c^ Since the severity of larval feeding damage in 2014 developed slowly from 1 to 4 weeks after seedling emergence, they are not presented in the table. ^d^ Cabbage treatment was not included in 2014.

## Data Availability

The data presented in this study are available on request from the corresponding author.
